# Nurse-led, telephone-based secondary preventive follow-up benefits stroke/TIA patients with low education: a randomized controlled trial sub-study

**DOI:** 10.1186/s13063-018-3131-4

**Published:** 2019-01-15

**Authors:** Anna-Lotta Irewall, Joachim Ögren, Lisa Bergström, Katarina Laurell, Lars Söderström, Thomas Mooe

**Affiliations:** 1 Department of Public Health and Clinical Medicine, Östersund, Umeå University, Umeå, Sweden; 2 Department of Pharmacology and Clinical Neuroscience, Östersund, Umeå University, Umeå, Sweden; 3 Unit of Research, Development and Education, Region Jämtland Härjedalen, Östersund Hospital, Östersund, Sweden

**Keywords:** Secondary prevention, Stroke, Transient ischemic attack, Socioeconomic position

## Abstract

**Background:**

The objective of this study was to analyze the impact of two forms of secondary preventive follow-up on the association between education level and levels of blood pressure (BP) and low-density lipoprotein cholesterol (LDL-C) after stroke/transient ischemic attack (TIA).

**Methods:**

We included a population-based cohort of 771 stroke and TIA patients randomly assigned (1:1) to secondary preventive follow-up within primary health care (control) or nurse-led, telephone-based follow-up (intervention) between January 1, 2010, and December 31, 2013, as part of the NAILED (nurse-based age-independent intervention to limit evolution of disease) stroke risk factor trial. We compared BP and LDL-C levels 12 months after hospital discharge in relation to education level (low, ≤10 years; high, >10 years) separately for the intervention and control groups.

**Results:**

Among controls, systolic BP (SBP) decreased only among the highly educated (−2.5 mm Hg, 95% confidence interval (CI) −0.2 to −4.8), whereas LDL-C increased in the low-education group (0.2 mmol/L, 95% CI 0.1 to 0.3). At 12 months, controls with low education not more than 70 years of age had higher SBP than controls of the same age with high education (5.8 mm Hg, 95% CI 1.0 to 10.6). In contrast, SBP in the intervention group decreased similarly regardless of education level, LDL-C decreased among those with low education (−0.3 mmol/L, 95% CI −0.2 to −0.4) and, in the subgroup not more than 70 years old, low-educated participants had lower LDL-C at 12 months than those with high education (0.3 mmol/L, 95% CI 0.1 to 0.5).

**Conclusions:**

Nurse-led, telephone-based secondary preventive follow-up led to comparable improvements in BP across education groups, while routine follow-up disfavored those with low education.

**Trial registration:**

ISRCTN Registry ISRCTN23868518, June 19, 2012 - Retrospectively registered.

## Background

Patients with a previous stroke or transient ischemic attack (TIA) are at high risk of subsequent cardiovascular events, including stroke [1–4], myocardial infarction [2, 3, 5], and cardiovascular death [2, 3]. Based on clinical trials, secondary preventive treatment with antihypertensives [6] and statins [7] decreases the risk of cardiovascular events after stroke and TIA. However, observational studies have shown that recommended treatment targets for blood pressure (BP) and low-density lipoprotein cholesterol (LDL-C) are often not met [8–10], indicating room for improvement in clinical practice.

Socioeconomic position (SEP), referring to social and economic factors that influence what position individuals and groups hold within the structure of society [11, 12], is an important risk marker for cardiovascular disease [13–19], including stroke [15–19]. Low SEP has been repeatedly associated with higher prevalence of cardiovascular risk factors [13, 20–22] and subclinical atherosclerosis [13, 20] as well as higher incidence of stroke [15–19] and other cardiovascular events [13–15]. In addition, several studies have reported a higher prevalence of cardiovascular risk factors among stroke patients with low SEP [17–19], and some evidence indicates an increased risk of stroke recurrence [17]. To effectively reduce the recurrence of cardiovascular events, secondary preventive treatment should be optimized across all socioeconomic groups. Whether control of BP and LDL-C after stroke/TIA differs based on SEP is currently unknown.

The randomized controlled NAILED (nurse-based age-independent intervention to limit evolution of disease) stroke risk factor trial investigates whether further optimization of secondary preventive care can be achieved through systematic, nurse-led, telephone-based follow-up including titration of medication [23]. According to published results, the NAILED intervention reached significantly lower levels of systolic BP (SBP) and LDL-C than follow-up according to standard care 12 months after hospital discharge [24]. The objective of the present study was to analyze the impact of these two forms of secondary follow-up on a possible association between education level and BP and LDL-C levels 12 months after stroke/TIA. We hypothesized that the telephone-based follow-up would achieve more equal outcomes across education levels than follow-up according to standard care.

## Methods

### Study design

This was an open randomized (1:1) controlled trial sub-study and a post-hoc analysis.

### Outcomes and sample size

The primary outcome was the mean difference in SBP between participants with high and low education (as defined below) 12 months after hospital discharge. We analyzed the following variables as secondary outcomes: the mean differences in diastolic blood pressure (DBP) and LDL-C levels between education groups at 12 months and changes in the SBP, DBP, and LDL-C levels between baseline and 12 months within each education group. We analyzed all outcome variables separately for the intervention and control groups. BP was measured in the sitting position after 5 min of rest. LDL-C was calculated from serum concentrations of cholesterol and fasting triglycerides by using the Friedewald formula. Based on a power calculation, study groups of 180 participants—standard deviation (SD) 19 mm Hg, mean SBP 140 versus 135 mm Hg, two-tailed alpha 0.05, and power 80%—were necessary to reliably detect a difference of 5 mm Hg in the mean SBP.

### Study population and setting

The study included participants from the NAILED stroke risk factor trial who remained in the trial and had measurement data available from the 12-month follow-up (*n* = 771). Recruitment for the NAILED stroke risk factor trial occurred at Östersund Hospital, which is the only hospital in the county of Jämtland, Sweden. In 2010, the county of Jämtland had 126,691 inhabitants, 35.0% of whom lived in the centrally located city of Östersund, 23.0% in villages of 500–4000 inhabitants, and the remaining 42.0% in the more sparsely populated surroundings. The mean age of the county’s inhabitants was 43.1 years and 50.0% were female. Among inhabitants 65 years or older, 46.3% had completed no more than 9–10 years of school and the remaining 53.7% had completed higher levels of education [25].

In Sweden, public inpatient and outpatient health care is provided to all citizens at a low cost but is not entirely free of charge. There is also a national system for stepwise subsidizing of medical drugs [26]. During the inclusion period, the hospital stroke unit generally treated admitted stroke and TIA patients, or another hospital care unit did if no bed was available in the stroke unit. The treating physicians initiated secondary preventive treatment in-hospital and, in some cases, followed by a return visit to the hospital out-patient clinic. Thereafter, primary health care was the principal responsibility of the secondary preventive follow-up. In primary health care, patients with a previous stroke or TIA are not routinely called for regular control of BP and blood lipids unless they are also diabetic. Medical prescriptions are renewed at the patient’s request and patients are welcome to book an appointment with a doctor if they want to discuss secondary preventive treatment further. At many primary health-care centers, patients can also visit at any time during opening hours for a self-measured BP check-up; results are later reviewed by a nurse or physician or both.

Since 1962, compulsory schooling in Sweden has consisted of 9–10 years of elementary school followed by 2–4 years of optional upper secondary school/vocational school leading to a vocational degree or qualifying for college and university studies. All levels of public school are free of charge. During the preceding three decades, the school system was more complex; compulsory school ranged from 6 to 8 years and was free of charge, but qualification for upper secondary school and university required graduation from a partly parallel school system. Altogether, 9–10 years of school was required to proceed to upper secondary school and thereafter university [27].

### The NAILED stroke risk factor trial

**Fig. 1:**
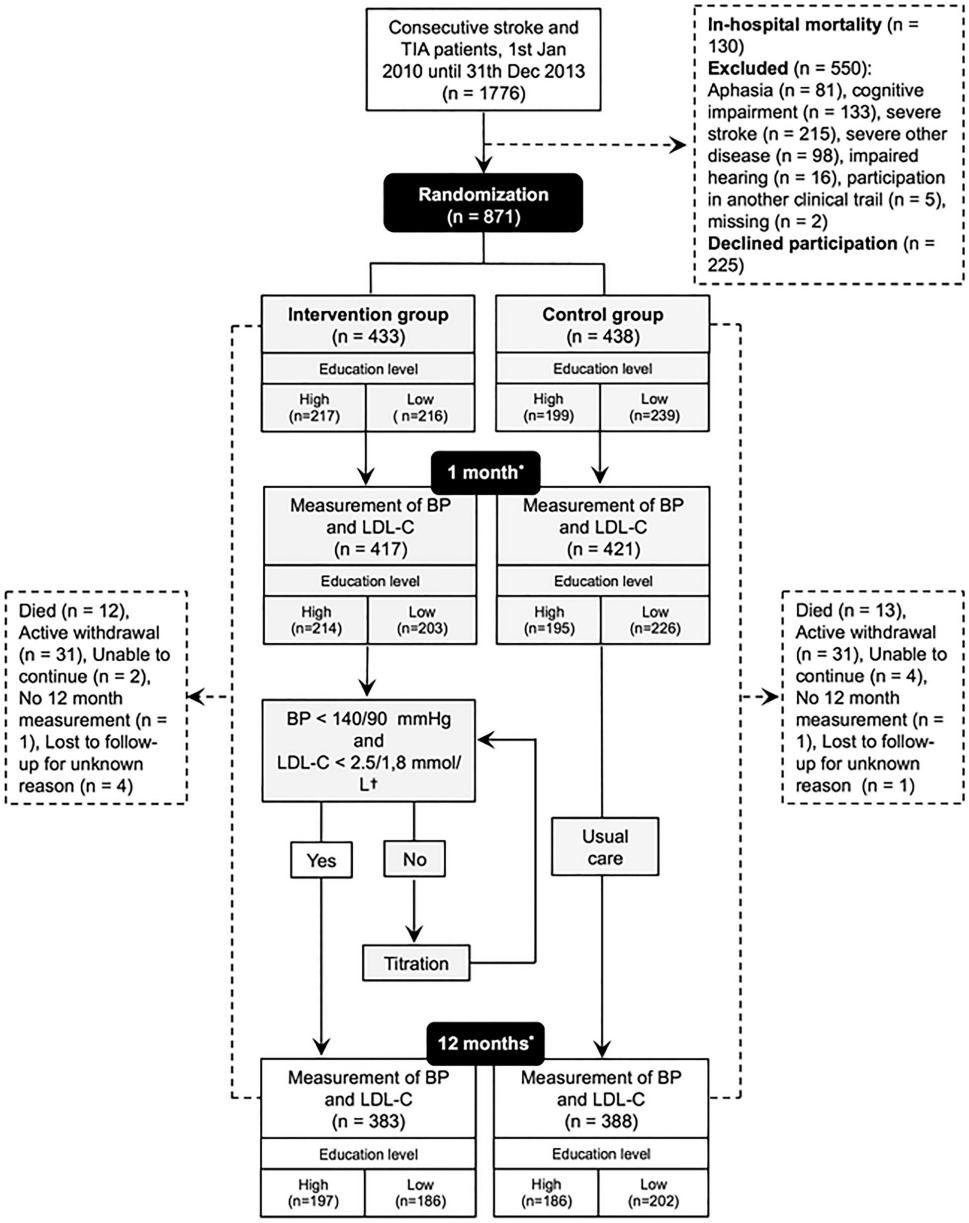
Participant flowchart. Abbreviations: *BP* blood pressure, *LDL-C* low-density lipoprotein cholesterol, *TIA* transient ischemic attack. ^*^After hospital discharge. ^†^Owing to an update to the local guidelines, the LDL-C target value was less than 1.8 mmol/L for participants with diabetes mellitus who had their 1-month measurement after March 31, 2013 (*n* = 20).

The NAILED stroke risk factor trial is an open, population-based, randomized controlled trial testing the hypothesis that nurse-led, telephone-based secondary preventive follow-up is more effective than standard-care follow-up in regard to achieving improved modifiable risk factor levels after stroke or TIA. The trial inclusion period was from January 1, 2010, to December 31, 2013, and follow-up is ongoing. Mooe et al. previously published the methods of the NAILED stroke risk factor trial [23]. Briefly, the trial recruited consecutive patients admitted to Östersund Hospital because of stroke or TIA (Fig. 1). Patients who were physically and cognitively able to participate in the study follow-up provided informed consent prior to inclusion. Exclusion criteria were participation in concurrent trials and/or an inability to participate because of impaired hearing, aphasia, cognitive impairment, or severe, often terminal, disease. After random assignment (allocation ratio 1:1; allocation sequence computer-generated in blocks of four; stratified for sex and the degree of disability according to the modified Rankin Scale, mRS), participants underwent nurse-led, telephone-based follow-up (intervention) or follow-up in accordance with local standard procedure (that is, mainly within primary health care (control)). A study nurse telephoned all participants for follow-up at 1 and 12 months after hospital discharge. The study nurses made repeated attempts to get in contact with participants who did not answer the first telephone call. Before each follow-up occasion, the participants had their BP measured and a blood sample for lipids taken at their closest health-care facility. Intervention follow-up included information about measurement results, lifestyle counseling (physical activity, diet, and smoking cessation), and assessment of pharmacological treatment. Assessments of lipid lowering treatment were restricted to participants with ischemic stroke or TIA. Participants in the intervention group who did not meet treatment targets for BP (<140/90 mm Hg) or LDL-C (<2.5/1.8 mmol/L) underwent pharmacological titration with repeated measurement and adjustment every 4 weeks until they achieved the treatment targets or they could achieve no further improvement. A study physician consulted on all pharmacological adjustments. Owing to an update to the local guidelines, the LDL-C treatment target for patients with diabetes changed during the course of the study. Consequently, for participants with diabetic mellitus who had their 1-month follow-up after March 31, 2013, (*n* = 20), the treatment target was LDL-C less than 1.8 mmol/L, but it was less than 2.5 mmol/L for the rest of the population.

Telephone contact with participants in the control group did not include any lifestyle counseling or changes to pharmacological treatment. BP and LDL-C measurement results were forwarded to the patient’s general practitioner for assessment.

### Data collection and definition of variables

All data presented in this report were from the NAILED stroke risk factor trial. The NAILED study team collected patient characteristics at baseline, including age, education level, functional level according to the mRS, cardiovascular risk factors, and medical history, in-hospital through patient interviews and a review of medical records. The study team also measured weight and height to calculate body mass index (BMI).

We used a dichotomized classification of education level. We defined low education as not more than 10 years of formal education and high education as the completion of more than 10 years of formal education.

The qualifying events, prior vascular events, and comorbid conditions refer to diagnoses made by clinical physicians. Stroke included both ischemic and hemorrhagic events with the exception of subarachnoid hemorrhage. We defined prior ischemic heart disease as including previous acute myocardial infarction, percutaneous coronary intervention, or coronary artery bypass grafting or a combination of these.

### Statistical analysis

Baseline characteristics are presented for the study population as a whole and for subgroups defined by education level. We performed between-group comparisons of baseline characteristics by using independent sample *t* tests for continuous variables and chi-squared test for categorical variables.

We analyzed differences in BP and LDL-C levels separately for the intervention and control groups. We used the paired sample *t* test to calculate the mean changes in SBP, DBP, and LDL-C between 1 and 12 months after hospital discharge. We estimated the mean differences in SBP, DBP, and LDL-C at 12 months by using general linear models. The initial model included sex and baseline characteristics significantly associated with education level. We then reduced the model to include only significant variables. Owing to high correlation with education level, age could not be included in the models; so we performed age-stratified analyses using a dichotomous classification of age with 70 years chosen as the cutoff based on the population mean. Owing to the low number of participants in each education group, this analysis did not include covariates. Participants with hemorrhagic stroke as the qualifying event were not included in analyses regarding LDL-C.

When applicable, missing data are indicated in the tables. We adopted a significance threshold of *P* = 0.05 for analyses using SPSS software, version 22.0 (IBM Corporation, Armonk, NY, USA).

## Results

The flow of participants is illustrated in Fig. 1. The mean age of participants (*n* = 771) was 70.7 (SD 10.6) years, 41.1% (*n* = 317) were women, and stroke was the more common qualifying event compared with TIA (61.0% versus 39.0%). Roughly half of the participants (50.3%, *n* = 388) had a low level of formal education (Table 1). Except for a higher occurrence of previous ischemic heart disease among men (14.8% versus 6.6%, *P* <0.001), the baseline characteristics of male and female participants, including education level, did not differ significantly (data not shown).

**Table 1:** Baseline characteristics and unadjusted associations with educational level

	All	Education level		*P* value*
		High	Low	
N (%)	771	383 (49.7)	388 (50.3)	
Age, mean (SD)	70.7 (10.6)	67.1 (10.9)	74.3 (9.2)	<0.001
Female, N (%)	317 (41.1)	151 (39.4)	166 (42.8)	0.343
Treatment group				
Intervention, N (%)	383 (49.7)	197 (51.4)	186 (47.9)	0.331
Control, N (%)	388 (50.3)	186 (48.6)	202 (52.1)	
Qualifying event				
TIA, N (%)	301 (39.0)	165 (43.1)	136 (35.1)	0.022
Stroke, N (%)^†^	470 (61.0)	218 (56.9)	252 (64.9)	
mRS score >2, N (%)	88 (11.4)	34 (8.9)	54 (13.9)	0.028
Current smoker, N (%)	106 (13.7)	53 (13.8)	53 (13.7)	0.943
BMI, mean (SD)^‡^	27.0 (4.5)	26.7 (4.3)	27.3 (4.6)	0.045
AF, N (%)	129 (16.7)	63 (16.4)	66 (17.0)	0.835
IHD, N (%)	88 (11.4)	25 (6.5)	63 (16.2)	<0.001
Previous stroke, N (%)^§^	95 (12.3)	31 (8.1)	64 (16.5)	<0.001
Diabetes, N (%)	128 (16.6)	47 (12.3)	81 (20.9)	0.001
CHF, N (%)	28 (3.6)	8 (2.1)	20 (5.2)	0.023
PAD, N (%)	22 (2.9)	10 (2.6)	12 (3.1)	0.688
Hypertension, N (%)	458 (59.4)	200 (52.2)	258 (66.5)	<0.001
Antihypertensives, N (%)^| |^	585 (76.2)	269 (70.6)	316 (81.7)	<0.001
1	215 (36.8)	108 (40.1)	107 (33.9)	
2	205 (35.0)	80 (29.7)	125 (39.6)	
≥3	165 (28.2)	81 (30.1)	84 (26.6)	
Lipid-lowering agent, N (%)^| |^	605 (78.8)	301 (79.0)	304 (78.6)	0.879
Simvastatin	487 (80.5)	248 (82.4)	239 (78.6)	
Atorvastatin	109 (18.0)	49 (16.3)	60 (19.7)	
Other	9 (1.5)	4 (1.3)	5 (1.6)	
Antiplatelet drug, N (%)^| |^	619 (80.6)	308 (80.8)	311 (80.4)	0.867
Warfarin, N (%)^| |^	116 (15.1)	54 (14.2)	62 (16.0)	0.475

Abbreviations: *AF* atrial fibrillation, *BMI* body mass index, *CHF* congestive heart failure, *IHD* ischemic heart disease, *mRS* modified Rankin Scale, *PAD* peripheral artery disease, *SD* standard deviation, *TIA* transient ischemic attack

^*^High versus low

^†^Intracerebral hemorrhage accounted for 14 (6.2%) and 9 (3.6%) of the qualifying stroke events in the highly educated and low-educated groups, respectively. The remaining qualifying stroke events were ischemic.

^‡^One missing value in the high-education group

^§^Prior to the qualifying event

^| |^One month after hospital discharge. Two and one missing values from the high- and low-education groups, respectively

Compared with the 771 participants, patients who did not remain in the trial at the 12-month follow-up (*n* = 100, Fig. 1) were older and more commonly female, had lower BMI and higher occurrence of atrial fibrillation and congestive heart failure, and a higher proportion had an mRS score of more than 2 and low level of education (data not shown).

### Baseline characteristics according to education level

**Fig. 2:**
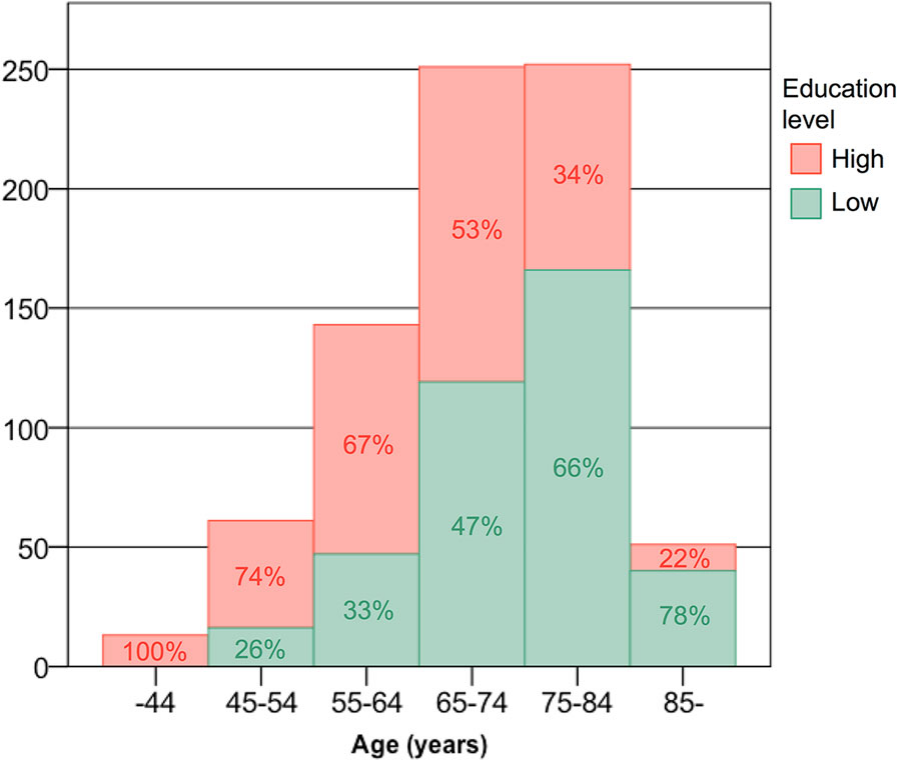
Education distribution according to age group

The proportion of participants considered to have a high education level decreased gradually with increasing age (Fig. 2). The pattern was the same for both men and women (data not shown). Consequently, participants with low education were older than participants with high education (Table 1). Compared with participants with high education, those with low education were more likely to be included in the study because of stroke and a higher proportion had an mRS score of at least 3. In addition, we found an association between low education and a higher prevalence of comorbid conditions and cardiovascular risk factors, including previous diagnosis of ischemic heart disease, previous stroke, congestive heart failure, hypertension, and diabetes mellitus.

### Baseline treatment and levels of BP and LDL-C

Baseline SBP and LDL-C levels measured 1 month after hospital discharge did not differ according to education level (Table 2). Pharmacological treatment at the same time point is shown in Table 1. Treatment with antihypertensives was more common among participants with low education, whereas the proportions prescribed warfarin, antiplatelet drugs, or lipid-lowering agents were not different.

**Table 2:** Association between education level and SBP, DBP, and concentration of LDL-C

	Intervention group			Control group		
	Education level		Between-group difference (95% CI)	Education level		Between-group difference (95% CI)
	High	Low		High	Low	
SBP	*N* = 197	*N* = 186		*N* = 186	*N* = 202	
1 month, mean (SD)^*^	135.9 (19.4)	138.2 (15.4)	NS	136.1 (17.9)	138.3 (19.7)	NS
12 months, mean (SD)	130.9 (17.9)	133.5 (15.6)	NS	133.6 (16.6)	137.8 (17.7)	4.2 (0.8 to 7.6)
Mean change between 1 and 12 months (95% CI)	−4.9 (−2.1 to −7.8)	−4.8 (−1.9 to −7.6)		−2.5 (−0.2 to −4.8)	NS	
DBP	*N* = 197	*N* = 186		*N* = 186	*N* = 202	
1 month, mean (SD)^*^	81.8 (12.1)	79.2 (11.3)	2.6 (0.2 to 5.0)	81.9 (11.4)	78.9 (10.4)	3.0 (0.8 to 5.2)
12 months, mean (SD)	78.9 (10.6)	77.2 (10.3)	NS	80.8 (10.3)	79.8 (10.1)	NS
Mean change between 1 and 12 months (95% CI)	−2.9 (−1.0 to −4.8)	−2.1 (−0.4 to −3.7)		NS	NS	
LDL-C^†^	*N* = 190	*N* = 182		*N* = 179	*N* = 197	
1 month, mean (SD) ^‡^	2.5 (0.8)	2.5 (0.8)	NS	2.4 (0.8)	2.4 (0.8)	NS
12 months, mean (SD)^§^	2.4 (0.9)	2.2 (0.7)	0.2 (0.1 to 0.4)	2.5 (0.8)	2.6 (1.0)	NS
Mean change between 1 and 12 months (95% CI)	NS	−0.3 (−0.2 to −0.4)		NS	0.2 (0.1 to 0.3)	

Abbreviations: *CI* confidence interval, *DBP* diastolic blood pressure, *LDL-C* low-density lipoprotein cholesterol, *NS* not significant, *SBP* systolic blood pressure, *SD* standard deviation

^*^In the control group, two values missing for each education level. In the intervention group, one value missing in the high-education group

^†^The LDL-C analyses did not include participants with hemorrhagic stroke as the qualifying event.

^‡^In the control group, four and two values missing in the high- and low-education groups, respectively. In the intervention group, one value missing in the high-education group

^§^In the control group, two and four values missing in the high- and low-education groups, respectively. In the intervention group, three values missing from each education level

### BP and LDL-C levels 12 months after hospital discharge

**Fig. 3:**
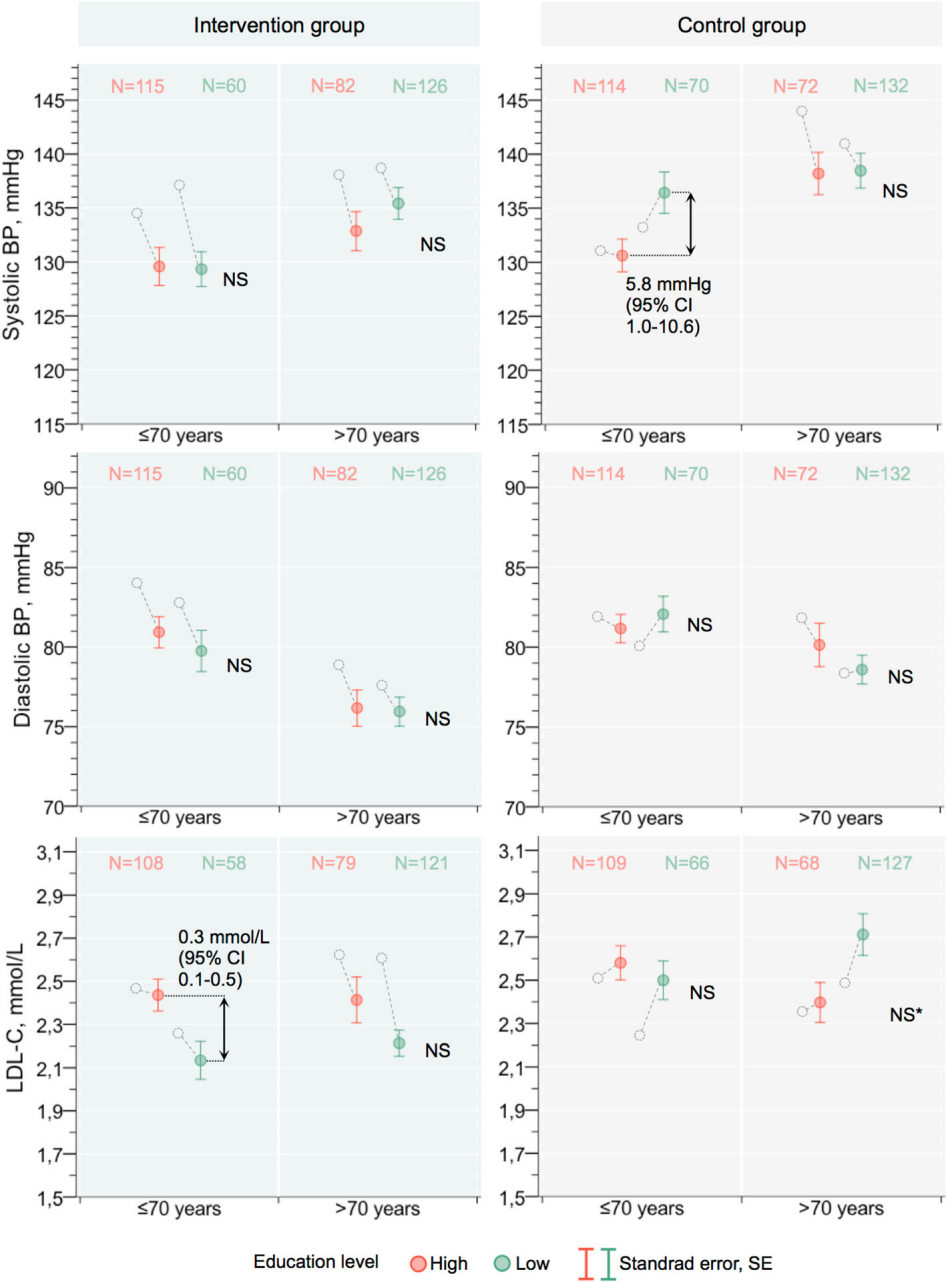
Age-stratified unadjusted association between education level and systolic blood pressure, diastolic blood pressure, and LDL-C 12 months after hospital discharge. The gray circles mark the corresponding levels at 1 month for each group. Abbreviations: *BP* blood pressure, *CI* confidence interval, *LDL-C* low-density lipoprotein cholesterol, *NS* not significant. ^*^Borderline significance: mean difference 0.3 mmol/L (95% CI −0.13 to 0.579, *P* = 0.061)

The association between education level and BP and LDL-C levels differed according to secondary preventive follow-up and age group. At 12 months, controls with low education had higher SBP than controls with high education (mean difference 4.2 mm Hg, 95% confidence interval (CI) 0.8 to 7.6; Table 2). After stratification for age, a significant difference in SBP between the high- and low-education subgroups remained only for those not more than 70 years of age (Fig. 3). In the intervention group, we found no association between education level and BP at 12 months (Table 2), regardless of age group (Fig. 3).

In regard to the change in BP between 1 and 12 months, SBP and DBP improved in the intervention group regardless of education level (Table 2). Mean SBP improved in controls with high education, but we found no change among controls with low education.

LDL-C at 12 months did not differ according to education level in the control group (Table 2), regardless of age group. However, we found an association between low education and an increase in LDL-C between 1 and 12 months (Table 2). We observed the opposite situation in the intervention group; an association of low education with lower LDL-C at 12 months (mean difference 0.2, 95% CI 0.1 to 0.4) resulted from a decrease in LDL-C in the low-education subgroup between 1 and 12 months (Table 2). After stratification for age, the difference at 12 months was significant only for participants in the intervention group not more than 70 years of age (Fig. 3). Notably, we found similar LDL-C levels across all groups with high education at 12 months and levels did not improve among highly educated participants during the first year, regardless of secondary preventive follow-up (Table 2 and Fig. 3).

Adjusting for sex, BMI, type of qualifying event, medical history, and functional level according to mRS score did not change the education level differences in SBP, DBP, and LDL-C levels at 12 months (data not shown).

## Discussion

In the present study, we found an association between low education and less favorable changes in SBP and LDL-C levels the first 12 months after stroke/TIA among participants receiving secondary preventive follow-up according to standard care. In the younger half of the population, we found an association between low education and higher SBP at 12 months. Participants who received follow-up according to the NAILED intervention program, however, achieved equal improvement of SBP across education levels, and we found no differences in BP according to education level at 12 months. LDL-C levels differed slightly in the intervention group at 12 months but in favor of those with low education. We found no significant improvement in LDL-C during the first year after stroke/TIA among highly educated participants, regardless of secondary preventive follow-up group.

The NAILED stroke risk factor trial is a population-based study in which a fairly small proportion of the eligible patients declined participation, and few ended their participation during the first year of follow-up. Therefore, the study population is representable of the general stroke and TIA population that could participate in this relatively simple form of secondary preventive follow-up. However, as social structure, health-care systems, and the organization of secondary preventive follow-up vary between regions and countries, the results of the present study may not be directly reproducible everywhere. In order to facilitate an evaluation of the external relevance of our conclusions in other populations, we have tried to describe the local conditions in as detailed a manner as possible.

We chose education as an indicator of SEP because it often reflects the conditions under which an individual was brought up while also often predicting future occupation, income, and related social context. Education level may also affect a person’s receptiveness to health education messages, communication with health-care providers, and the utilization of health-care resources [12]. However, the education level of the overall population has increased over time with gradual changes in the importance and value of education. Unfortunately, the size of the study population limited analysis to binary classification of education, and stratification into more narrow age groups was not possible. As no validated standard is available for creating groups of relative education levels, we chose the same cutoff level for high and low education regardless of age. This may explain why an association between education level and outcome at 12 months was found only among participants not more than 70 years of age.

Non-communicable diseases (NCDs), including stroke, have been addressed by the World Health Organization as major threats to global health, disproportionally affecting low- and middle-income countries [28]. Low SEP is an important risk marker for stroke and other cardiovascular diseases also within high-income countries [13–19], and many studies report social stratification in the prevalence of cardiovascular risk factors [13, 17–22]. In regard to secondary preventive care after stroke/TIA, studies of socioeconomic differences are limited to prescription of [29–33] and adherence to [29, 34, 35] secondary preventive drugs and results vary between studies. In studies from Ontario [29] and Vienna [30], socioeconomic variables were not associated with any substantial differences in prescription of secondary preventive drugs at discharge. On the contrary, data from the national Swedish stroke register showed that (1) low-educated patients with ischemic stroke and atrial fibrillation were less likely to be initiated on oral anticoagulation treatment (2009–2012) [33] and that (2) ischemic stroke patients with a low level of education or income were less likely to be prescribed a statin [32]. Similarly, a study from the South London Stroke Register (1995–2010) found differences in proportions treated with oral anticoagulants at 3 months after discharge [31]. In this study, we found no differences in the prescription of oral anticoagulants or lipid-lowering drugs at 1 month after hospital discharge. It may be that such socioeconomic differences in secondary preventive treatment have diminished over time, but it should also be considered that our data come from a selected cohort which is not directly comparable to unselected register data. We are not aware of any previous study analyzing socioeconomic differences in BP and LDL-C levels during follow-up after stroke/TIA; to the best of our knowledge, this study is the first to demonstrate a favorable impact of an intervention follow-up strategy on reducing such socioeconomic differences.

Based on clinical trials, the difference in SBP observed between participants with low and high education, not more than 70 years of age, in the NAILED control group is large enough to be of clinical relevance regarding the risk of subsequent cardiovascular events [6]. In the “Global action plan for the prevention and control of NCDs 2013-2020”, one of the goals is “to strengthen and orient health systems to address the prevention and control of NCDs and the underlying social determinants through people-centered primary health care and universal health coverage” [36]. The Swedish health-care system includes universal coverage and subvention of medication for all citizens. However, our results indicate that secondary preventive care according to standard procedure does not achieve equal results across education levels, at least not among the younger half of the population. Based on our results, a standard-care strategy in which the intensity of follow-up depends largely on the patient’s own initiative seems to benefit highly educated participants more than those with a lower level of education. On the other hand, our results are encouraging because they suggest that socioeconomic inequality in secondary prevention can be reduced through a relatively simple reorganization of the follow-up strategy, including an outreach approach, regular BP and blood lipid check-ups, and telephone-based, systematic adjustment of medication and lifestyle counseling. This is in line with previous research which has shown that low education is associated with low health literacy, a concept that may include lower ability to communicate and engage with health-care providers, lower up-take of information regarding health recommendations [37], and lower adherence to pharmacological treatment [38]. It is important to acknowledge that universal coverage of health care alone is not enough to eliminate social inequality in health outcomes. This may have implications for policy making regarding organization of secondary preventive follow-up after stroke, but the results need verification in repeated studies before firm conclusions can be made.

Notably, LDL-C levels were similar across all high-education groups at 12 months, and we found no significant improvement in LDL-C levels among highly educated participants during the first year, regardless of secondary preventive follow-up. A recently published registry-based study from Sweden reported lower adherence to statin treatment among highly educated participants the first 2 years after ischemic stroke [34]. Together with our findings, this report suggests that the implementation of optimized lipid-lowering treatment might be more challenging among the highly educated. At this point, we can only speculate about potential mechanisms underlying a possibly lower adherence among those more highly educated. However, it has been described that high education level can be associated with a higher receptiveness to information regarding health [37]. Paradoxically, this may decrease adherence to statin prescriptions since potential side effects have been highly debated in different popular media for several years. However, it should be noted that the changes and differences in LDL-C levels observed in our study were relatively small overall and have uncertain clinical importance.

Optimization of secondary preventive treatment across all socioeconomic groups is important to effectively reduce the recurrence of cardiovascular events, especially as low SEP may be associated with an elevated risk of stroke recurrence [17]. Socioeconomic differences in the recurrence of cardiovascular events after stroke do require further assessment, as evidence from unselected populations is lacking. Importantly, future trials of interventions to improve secondary preventive follow-up need to consider the effect of the intervention across socioeconomic groups as well as the representativeness of the study sample in terms of the participation rate in different socioeconomic groups. Also, secondary prevention of cardiovascular disease requires long-sightedness, and from that perspective, the 12-month follow-up in our study is a short period of time. It will be important for future studies to assess whether this strategy of follow-up is effective also during more long-term intervention and follow-up. Finally, it remains to be proven that an intervention that reduces socioeconomic differences in risk factor levels also reduces socioeconomic differences in the recurrence of cardiovascular events.

## Conclusions

In the studied population, the association between education level and BP and LDL-C levels the first 12 months after stroke/TIA depended on the secondary preventive follow-up. Nurse-led, telephone-based secondary preventive follow-up resulted in equal improvements in SBP across education groups and improved LDL-C levels among participants with low education. Our results indicate that socioeconomic differences in risk factor levels after stroke/TIA exist in standard care but can be reduced through systematic outreach with a relatively simple follow-up procedure.

## Abbreviations

BMI: Body mass index
BP: Blood pressure
CI: Confidence interval
DBP: Diastolic blood pressure
LDL-C: Low-density lipoprotein cholesterol
mRS: Modified Rankin Scale
NAILED: Nurse-based age-independent intervention to limit evolution of disease
NCD: Non-communicable disease
SBP: Systolic blood pressure
SD: Standard deviation
SEP: Socioeconomic position
TIA: Transient ischemic attack
